# Empirical validation of the psychological concept of a perceived feeling of ‘energy’: Advancement into the study of positive psychology

**DOI:** 10.1371/journal.pone.0259762

**Published:** 2021-11-18

**Authors:** Huy P. Phan, Bing H. Ngu, Si-Chi Chen, Ruey-Yih Lin, Hui-Wen Wang, Jen-Hwa Shih, Sheng-Ying Shi

**Affiliations:** 1 School of Education, University of New England, Armidale, Australia; 2 College of Education, National Taipei University of Education, Taipei, Taiwan; 3 Department of Industrial Engineering and Management Information, Huafan University, New Taipei City, Taiwan; 4 Department of Asian Philosophy and Eastern Studies, Huafan University, New Taipei City, Taiwan; 5 Department of Buddhist Studies, Huafan University, New Taipei City, Taiwan; University of Macau, MACAO

## Abstract

The *paradigm of positive psychology*, significant in nature, helps to explain the proactivity and motivation of human agency, such as a secondary school student’s state of autonomy, confidence, and personal resolve to strive for optimal learning and/or non-learning experiences. Our recent research development, in tandem with other scholars’ inquiries, has focused on one aspect of positive psychology–namely, a person’s achievement of ‘optimal best’, which reflects the maximization of his/her state of functioning (e.g., cognitive functioning). Capitalizing on our previous research, we develop a psychological concept that we term as a ‘perceived feeling of energy’. A perceived feeling of energy (e.g., a perceived feeling of liveliness) is proposed to act as a ‘motivational engine’, or as a central driver, which then could predict and enhance a person’s achievement of optimal best. Six hundred and twenty-seven university students (N = 438 women, 189 men) responded to a suite of self-report questionnaires. Structural equation modelling (SEM) techniques were used to test a conceptual model, where we focused on the *antecedent* (i.e., the direct impact of *self-efficacy* on a perceived feeling of energy) and *consequence* of a perceived feeling energy (i.e., the impact of a perceived feeling of energy on *personal resolve*, and the *sustaining* of optimal best). Analysis of results showed support for our original hypothesized model–for example: self-efficacy as an antecedent of energy and the central role of the energy as a predictor and potential mediator of future outcomes.

## Introduction

The *study of motivation*, situated within the context of academic learning, is constantly evolving. One interesting line of inquiry, in this case, relates to the development of theoretical orientations, pathways and means, opportunities, pedagogical practices, etc. that could encourage and promote ‘optimal’ learning and non-learning experiences. Optimal learning experience, commonly known as ‘optimal best’ [1, 2], coincides with the *paradigm of positive psychology* [3–5], and reflects the maximization of an internal state of functioning (e.g., a secondary school student’s optimal cognitive functioning in Calculus) [1, 6]. Optimal best differs from sub-optimal experiences, which are deficit and maladaptive, requiring remedy and the development of preventive measures. It is important in school settings and academic contexts that concerted efforts are made to help facilitate students’ optimal learning and non-learning experiences [6, 7]. Development of the *theory of human optimization* [1, 7, 8] has led to our recent conceptualization of a psychological concept, which we term as perceived ‘energy’, or ‘personal experience of energization’, for empirical research validation.

We rationalize that perceived experience of energization, positive in nature [7], could in fact facilitate and enhance the proactivity of human agency [9]. Within the context of academic settings, for example, proactivity of human agency may consist of a student’s state of motivation, personal resolve, and self-determination to strive for educational (e.g., mastery in a subject matter) and non-educational (e.g., positive social relationship) successes. We argue that energy, or perceived experience of energization, could actually serve as a central ‘optimizing driver’, which then would predict and/or account for achievement in optimal best. We acknowledge that conceptualization into the *operational nature of energy* is still in its early stage of evolution with potential caveats for revision, re-articulation, and/or ongoing development. The current study, empirical and a first, seeks to establish evidence into the operational nature of energy–that energy, shaped by *self-efficacy belief for academic learning* [9, 10], would predict *a state of personal resolve* [8, 11], giving rise to the achievement of optimal best [1, 12]. Our quantitative examination, via means of the use of structural equation modeling (SEM) techniques [13, 14], is significant in terms of providing insightful information for the purpose of educational practice and theoretical contribution [1, 7].

### Achievement of optimal best and the importance of positive psychology

One notable aspect of human agency [9, 10], situated within the context of schooling, relates to a student’s autonomy and his/her free choice to achieve a state of optimal best [1, 2, 12]. Experience of optimal best, or commonly known as *optimal functioning* [1, 2], is a non-deficit feat that reflects the *maximization* of a person’s internal state of functioning–for example, within the context of academic learning, optimal cognitive functioning may indicate a student’s ability to write a 5000-word essay and subsequently receiving an A^+^ grade for this for this effort. Non-academically, likewise, a state of optimal best may consist of a professional football player’s testament of his ability to score 25 goals for the forthcoming 2021/2022 season, or an employee’s optimal state of resilience, personal resolve, and motivation to overcome difficulties and to achieve exceptional KPIs [15, 16].

The study of optimal best, situated within the context of academic learning, is insightful and may provide relevant information pertaining to a student’s state of motivation (e.g., buoyancy) [17, 18], perceived experience of flourishing [19, 20], and positive or negative emotions [21, 22]. More importantly, of course, successful accomplishment of optimal best may also reflect an institution’s ethos in quality teaching and curriculum development. Indication of sub-optimal accomplishments, in contrast, would connote ineffective teaching, low quality curriculum development, and/or evidence of student disengagement [23, 24]. Our research interest of optimal best [1, 2, 12] relates to the *study of positive psychology* [3–5], which emphasizes remedy of maladaptive life conditions and the promotion of positive life conditions (e.g., a flourished state of functioning) [25]. Positive psychology, as the nomenclature suggests, coincides with the motivation and proactivity of human agency and explores a person’s and/or an organization’s optimal state of functioning [26]. In brief, according to Sheldon and colleagues [27], positive psychology is defined as:

“the scientific study of optimal human functioning. It aims to discover and promote the factors that allow individuals and communities to thrive. The positive psychology movement represents a new commitment on the part of research psychologists to focus attention upon the resources of psychological health, thereby going beyond prior emphases upon disease and disorder” (Section 2).

Fraillon’s [1] seminal report in the 2000s is significant for its brief theoretical account of optimal best and, importantly, the *process of human optimization*. According to Fraillon [1], there are two main levels of best practice or state of functioning: (i) ‘actual best’ (i.e., denoted as L_1_), which relates to what a person is capable of at present (e.g., I am capable of successfully solving equations with one unknown, *x*), and (ii) ‘notional best’ (i.e., denoted as L_2_), which is concerned with the person’s testament of his/her maximum capability (e.g., I believe I am capable of successfully solving equations with two unknowns, *x* and *y*). Interestingly, Phan and Ngu [28] recently updated Fraillon’s [1] nomenclatures to the following equivalencies:

- Actual best practice to ‘*recognition* of realistic best’ (i.e., the rationalization that one is able to recognize and attest to what one is capable of–for example: I recognize that I am capable of solving equations with one unknown, *x*).
- Notional best practice to ‘*belief* of optimal best’ (i.e., the rationalization that one may express his/her belief of what he/she is able to achieve at a maximum level–for example: I believe that I am capable of solving equations with two unknowns, *x* and *y*).

From the brief introduction outlined, a pervasive question that is noteworthy for consideration in school contexts entails the following: *how do we assist students to achieve an optimal state of cognitive functioning* [6, 16]? This question places strong emphasis on the development of appropriate pedagogical designs, educational programs, institutional policies, etc. that would serve to facilitate optimal learning and non-learning experiences [1, 2, 12]. By the same token, we contend that enhancing optimal learning and non-learning experiences could help negate and prevent sub-optimal schooling experiences [8], which indeed are maladaptive and detrimental. For example, according to Phan and his colleagues [8, 29], sub-optimal learning experiences in a subject matter would closely align with a low state of motivation and a high level of cognitive load imposition [30, 31].

### The concept of ‘energy’: A conceptualization for consideration

One of our research interests, to date, has involved the study of the optimization of optimal learning experiences in academic learning. The significance of existing research [e.g., 1, 7, 12, 28] has led to our recent development of an ‘optimizing’ concept, which we termed as ‘energy’ or, more appropriately, a ‘perceived feeling of energy’. We acknowledge, though, that this psychological concept is still preliminary in terms of conceptualization and research evolution. For example, in an earlier draft of this article, one of the reviewers pondered and asked whether our concept of energy was/is analogous to that of a state of motivation? Despite the reviewer’s reservation, however, we rationalize that our conceptualized concept is innovative and original, coinciding closely with the recent theory of human optimization [1, 7, 16] and the paradigm of positive psychology [3–5]. In particular, as Phan and his colleagues [7] recently explained, the theory of human optimization [1, 8] purports that there is some ‘buoyant force’, similar to an analogy of “matters passing through a water hose” [32, 33], which could activate to mobilize a person’s state of functioning, propelling an improvement and/or achievement from T_1_ to T_2_, etc. Indeed, we acknowledge that this analogy [7, 32, 33] is insightful as it provides logical grounding for the study of energy as an optimizing force, which could help explain a person’s achievement of optimal best.

*Philosophical psychology* is an interesting paradigm, which involves the use of philosophical reasoning, personal understanding, and intellectual intuition to assist in the conceptualization and development of new psychological concepts and/or relationships between psychological concepts [34]. There are some conceptualizations of theoretical concepts such as Buddhist mindfulness [35, 36] and spiritual and esoteric experiences (e.g., transcendence experience) [37, 38] that are purely philosophical, making it somewhat difficult to scientifically validate. In a similar vein, drawing from Fraillon’s [1] introduction and using philosophical psychology as a theoretical framework, we recently developed the *theory of human optimization* [7, 8, 16], which may help to explain the facilitation of achievement of optimal best. In this sense, how does a person, an organization, a community, etc. achieve an optimal state of functioning (e.g., optimal cognitive functioning)?

Fraillon’s [1] original conceptualization briefly described the term ‘optimization’, which details the improvement and/or progression of a person’s state of functioning (e.g., a student’s cognitive functioning) from T_1_ (i.e., L_1_) to T_2_ (i.e., L_2_). Achievement of L_2_ from L_1_ is testament of a person’s improvement, progression, and/or personal growth in a subject matter and, more importantly, indicate his/her ‘state of flow’ [39–41]. A state of flow, coinciding with optimal best or optimal functioning, according to Fraillon [1] and other researchers [6–8], requires some ‘optimizing force’ and/or buoyancy.

**Fig 1:**
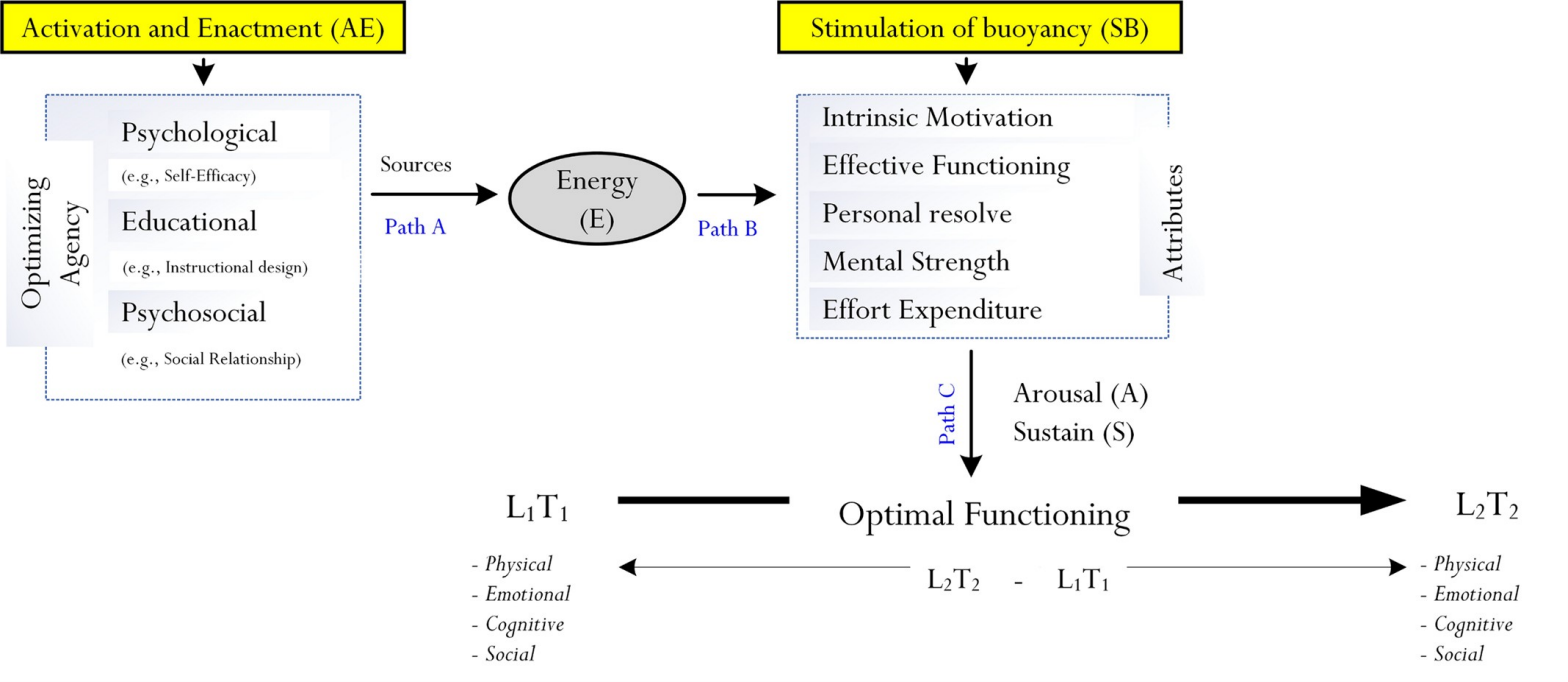
Proposed process of optimization. The process of optimization, as Phan, Ngu, and Yeung (2019) detailed in their recent publication, considers three fundamental aspects and, correspondingly, three pathways (i.e., Path A, Path B, and Path C): (i) the activation and enactment (denoted as ‘AE’) of optimizing agents, (ii) the instilment of a perceived feeling of energy, and (iii) the stimulation of buoyancy of different psychological attributes (e.g., intrinsic), which would arouse and sustain a state of functioning. L_**1**_ = current best practice, L_**2**_ = optimal best practice, T*1* = time 1, T2 = time 2. Source: Phan, H. P., Ngu, B. H., & Yeung, A. S. (2019). Optimization: In-depth examination and proposition. *Frontiers in Psychology*, 10(Article 1398), 1–16. doi:10.3389/fpsyg.2019.01398.

In their recent refinement and update of the theory of human optimization [1, 8], Phan and his colleagues [7, 16], philosophically, considered energy as a central ‘motivational driver’ or mechanism, which could explain a person’s improved state of functioning from T_1_ to T_2_. In a similar vein, as shown in Fig 1, we propose here that energy, or a perceived feeling of energy, could govern and ‘propel’ a person to strive for improvement and/or progression (i.e., L_1_ → L_2_). Empirical validation of this conceptualization, via correlational means would help advance the study of human optimization and the achievement of optimal best [e.g., 1, 7, 12]. Referring to our earlier example, a football player’s desire to achieve optimal best (i.e., to score 25 goals for the forthcoming 2021/2022) would require some form of energy or optimizing force, such as him/her having a healthier diet, an improved methodological training technique, the use of repeated practice, etc. Our proposition offers an alternative and/or additional viewpoint, focusing on the impact of energy: that a perceived feeling of energy, via different means (e.g., encouraging feedback from an external source) would intricately link to and/or initiate other sub-psychological processes (e.g., increased persistence and mobilization of effort), resulting in the football player’s successful striving to improve and/or progress from L_1_ to L_2_.

### A state of energy: Conceptualization of a definition

How do we define energy? We often associate the word energy with science, especially in the area of physics where it has different meanings and types–for example: nuclear energy, thermal energy, and mechanical energy (Source: https://www.britannica.com/science/energy). From a general point of view, energy may associate with the following: ‘doing work’, ‘a motion’, ‘a force’, and ‘effort’. Initially, aside from human optimization [e.g., 1, 7, 12], we used the positive psychology literature [3–5] to justify our proposition for the inclusion of such concept. With the emergence in research interest in the area of positive psychology [3–5], a number of researchers have proposed comparable psychological concepts that may assist to explain a person’s achievement of optimal best–for example, *buoyancy* [42, 43], *thriving* [44, 45], *personal striving* [6, 46], and *energy* [7, 47]. These comparable concepts are distinct but share a point of commonality in terms of their nature and characteristics–namely, that they are positive and proactive, helping to improve performance and/or individual progress.

Unlike buoyancy, thriving, and personal striving, research inquiry into the operational nature of energy has been relatively scant. Aside from recent development in philosophical reasoning of optimization [7, 47], very little, if any, consideration into the complex nature of energy has been made. To advance our initial conceptualization and definition, we sought some assistance from some of our third-year undergraduate students who were enrolled in a course that we taught at the time (and still teach). In one of our tutorial workshops, which focused on the module of *measurement*, *assessment*, *and evaluation* [48–50], we informally posed a tutorial task for a 40-minute in-class discussion: to explore and to consolidate a basic definition of ‘energy’ with reference to the study of positive psychology [3, 5, 27], and to consider a Likert measure and/or a survey that could assess this psychological concept. Our intention, in this analysis, was to introduce students to the concept of *measurement and assessment*, via means of use of both Likert-scale questionnaires and open-ended surveys. Interestingly, after a whole class discussion, we were able to identify and collectively conclude some common keywords for the concept of energy: ‘liveliness’, ‘vitality’, ‘inner strength’, ‘stamina’, ‘vigor’, ‘endurance’, ‘excitement’, ‘adrenaline’, and ‘buoyancy’.

By all account, the mentioned keywords (e.g., stamina, vigor) are similar to each other in terms of their nature and characteristics, leading us to surmise the possibility that energy is something that is ‘positive’, ‘driven’, and ‘dynamic’. In terms of contextualization then, energy may be considered as “…. a perceived feeling of liveliness, adrenaline, and mental strength, which could associate with different types of psychological processes and/or factors (e.g., effort expenditure) that would, in turn, improve the progression of a person’s state of functioning”. This proposed definition of energy connotes the importance of a person’s *perceived feeling* that he/she is ‘energized’, or ‘de-energized’, when faced with a contextual situation. We contend that, methodologically, the measurement and assessment of a person’s perceive feeling towards some known entity (e.g., perceived feeling about a person or about learning a topic) is logical and plausible (e.g., consider, say, the following: “I feel very excited about learning this topic in Calculus”). In this sense, the measurement and assessment of a student’s ‘perceived feeling’ of energy is valid and possible whereas, in contrast, measuring the complex nature of energy itself would be extremely difficult.

### Testing the operational nature of energy

**Fig 2:**
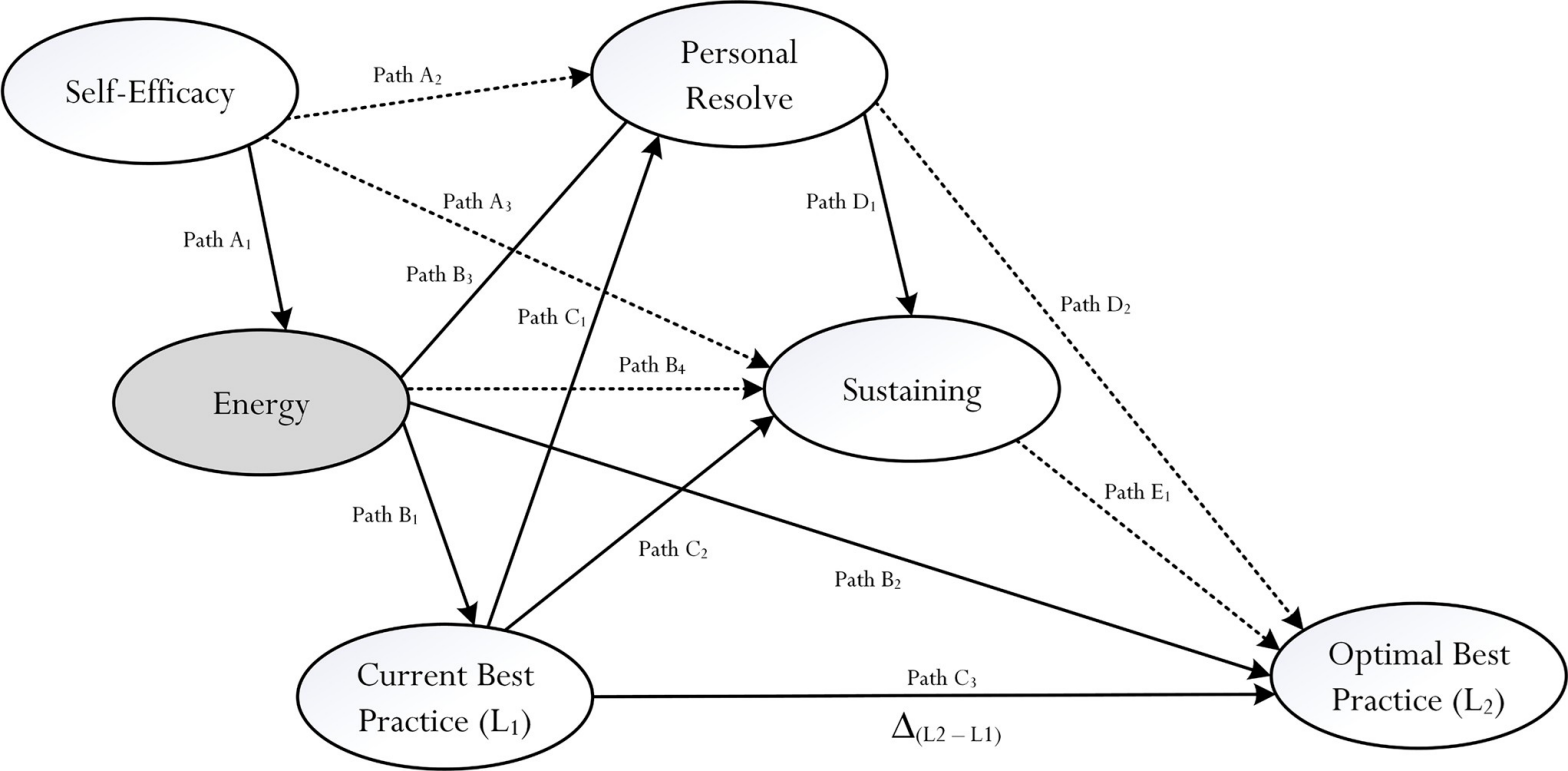
Conceptual model for investigation. Proposed conceptual model for statistical testing, which consists of a number of structural pathways (i.e., Path A_**1**_ –Path E_**1**_). According to Phan, Ngu, and Yeung (2019), successful experience of optimal best, L_**2**_, correspondingly associates with what is termed as ‘Δ_**(L2 –L1)**_’ (i.e., a ‘quantitative’ and/or ‘qualitative’ experience between current best practice and optimal best practice. The major paths for statistical testing and empirical validation are depicted as solid black arrows (e.g., Path A_**1**_, Path B_**1**_, etc.), whereas the minor, or lesser significant, paths are depicted as dotted black arrows (e.g., Path A_**2**_, Path A_**3**_, etc.).

The present study is innovative for its attempt to empirically explore the concept of energy, which may serve to account for the optimization of a person’s internal state of functioning [7, 16]. We rationalize, however, that measuring and assessing the true nature of energy itself with Likert-scale measures is somewhat limited and/or inaccurate. Likert-scale measures, from our point of view, are more appropriate in helping to measure and assess a person’s *perceived feeling* and/or *experience* of some known entity (e.g., a person’s perceived state of self-efficacy). In this sense, we cautiously argue that from a measurement point of view, the term of ‘a perceived feeling of energy’ is appropriate for usage and may, in fact, serve as a proxy indicator of the concept of energy itself. As depicted in Fig 2, we propose that energy or, more accurately, a perceived feeling of energy, would act as a central predictor and potential mediator of future adaptive outcomes (e.g., academic performance). Specifically, for consideration, we focus on three distinct inquiries that may advance our theoretical understanding of the process of optimization of optimal best: the *source* of a perceived feeling of energy, the *explanatory account* of energy, and the *sustained effect* of a perceived feeling of energy.

#### i. The source of energy

Our first proposed inquiry considers the *formation* of a perceived feeling of energy–that is, to identify and validate a source of information that could assist in the formation of a perceived feeling of energy. What is it that would facilitate and/or instill a person’s positive feeling of energy? There are a number of psychosocial factors and psychological variables that we believe are of relevance. One notable psychological variable is *self-efficacy* [9, 51], which is defined as “beliefs in one’s capabilities to organize and execute the courses of action required to produce given attainments” [9]. Why do we select self-efficacy [9, 51] for examination when, in fact, extensive literatures indicate that there are other potent psychological constructs (e.g., self-concept) [52, 53] as well? Self-efficacy, as existing research has shown, is a powerful predictor and mediator of different types of educational and non-educational outcomes [54, 55].

The explanatory nature of self-efficacy [56–59], consisting of a person’s improved persistence and effort expenditure, as well as the active mobilization of his/her effective responses and self-regulatory processes makes it a sound and relevant source of information for consideration. This examination (e.g., self-efficacy → energy, where ‘→‘ = predictive influence) is logical, contending that a heightened state of self-efficacy could initiate and instill a perceived level of energy for academic learning. Moreover, as a potential source of information, we postulate two contrasting possibilities: (i) self-efficacy, which is positive (i.e., a positive state of self-efficacy) and would instill a perceived feeling of energy *versus* (ii) inefficacy, which is negative (i.e., a negative state of self-efficacy) and would negate a perceived feeling of energy. This examination (i.e., a positive state of self-efficacy *versus* a negative state), indeed, could help provide clarity into a person’s achievement of optimal best, via means of self-efficacy and, hence, his/her perceived feeling of energy. Moreover, testament of evidence of a positive relationship between self-efficacy and a perceived feeling of energy could, in fact, support the aforementioned discussion–that a heightened state of self-efficacy could instill optimistic feelings and enriched experiences, giving rise to a comparable perceived level of energy, which then would assist a person to achieve an optimal level of best practice. In contrast, of course, a state of inefficacy, which is ineffective and detrimental, would negate the perceived feeling of energy (i.e., a person is unlikely, in this case, to perceive a feeling of energy), resulting in sub-optimal learning experiences.

#### ii. The positive effect of perceived feeling of energy

The premise of our conceptualization contends that a perceived feeling of energy (e.g., a person’s recall and indication of liveliness), as a central ‘driver’ of the process of optimization, could motivate a person to improve his/her state of functioning from T_1_ to T_2_. From a quantitative point of view, we reason that a positive predictive effect of a perceived feeling of energy, denoted as + β (i.e., beta), would indicate its potency. For example, in accordance with Fig 2, a positive β value from statistical analysis would illustrate the predictive effect of a perceived feeling of energy on different types of adaptive outcomes (i.e., energy → adaptive outcome, +ve β value). A negative β value or a non-statistically significant β value, in contrast, would suggest a perceived lack of energy.

Exploring the predictive power of a perceived feeling of energy may shed insightful information, which could help advance the study of optimization [1, 2, 8]. Our proposition considers whether and/or to what extent a student’s perceived feeling of energy could positively predict a comparable psychological construct, which we termed as ‘personal resolve’ [11, 46, 60]. Personal resolve, similar to the nature of *self-determination* [61, 62], is defined as a person’s ‘unwavering focus’ to stay on task without any indication of uncertainty and/or reservation. Moreover, personal resolve relates to a person’s conviction that his/her choice, positioning, and a course of action at a particular point in time are indeed correct, despite what others may say and/or advise (e.g., “Despite what my colleagues have advised, I strongly believe with conviction that I am on track….”) [6, 46]. In this sense, we contend that personal resolve espouses a number of life characteristics, such as a state of decisiveness, unchanging viewpoint, and mental fortitude to strive for success, regardless of perceived obstacles, difficulties, hardships, etc.

An interesting aspect for examination, as shown in Fig 2, is whether a perceived feeling of energy, in tandem with self-efficacy for academic learning [9, 54, 55], would act as a potent determinant of personal resolve. Previous research has found that a state of personal resolve is not naturally perpetuated, but instead relies on the prompting of different types of psychosocial factors and/or motivational processes [6, 46, 60, 63]. For example, in one of the earlier studies involving university students, Phan, Ngu, Shih, Lin, Shi, and Wang [46] found that self-efficacy for academic learning positively influenced a state of personal resolve (β = .83, *p* < .001). In a study that consisted of secondary school students, likewise, Phan and Ngu [6] observed that a student’s current knowledge in a subject matter (β = .17, *p* < .01) as well as his/her intention to strive for educational success (β = .43, *p* < .001) both served as sources of personal resolve. In a longitudinal study that focused on differing contextualized self-efficacy beliefs, Phan and his colleagues [11] noted that course-specific self-efficacy for academic learning at T_1_ exerted a statistically significant effect on personal resolve at T_2_ (β = .58, *p* < .001). Overall, then, extrapolating this line of evidence suggests that personal resolve towards a particular course of action (e.g., a student engages in academic learning of Calculus) requires some form of ‘initiation’ and ‘activation’. From our point of view, a perceived feeling of energy could instill a sense of ‘feel-good experience’, which then would act to strengthen a student’s personal resolve. In a similar vein, of course, a lack of energy (e.g., a lack of liveliness and vitality) could weaken a person’s feel-good experience, resulting in the negation of his/her state of personal resolve.

#### iii. The importance of a sustained effect: The use of perceived feeling of energy, personal resolve, and self-efficacy

In terms of optimal efficiency [64], it is important that a psychological concept of entity is able to sustain its potent effect (e.g., self-efficacy for academic learning sustains its predictive effect on performance outcome). In the context of schooling, for example, it is advantageous to have an educational program and/or a pedagogical practice that could predict and sustain its positive effect on an adaptive outcome (e.g., the positive effect of academic self-efficacy at T_2_ on critical thinking at T_4_, β = .14, *p* < .05) [65]. Instantaneous or a short-term effect (e.g., self-efficacy at T_1_ on critical thinking at T_1_, β = .16, *p* < .05) [66], in contrast, may indicate ineffectiveness and, consequently, serve very little, if any, meaningful purpose. Moreover, of course, instantaneous and/or short-term effects are ‘wasteful’, especially in in terms of expenditure of human capitals, cognitive resources, etc. In this sense, does a perceived feeling of energy have a long-term effect on a future adaptive outcome (e.g., T_1_ perceived feeling of energy → T_2_ personal resolve, where T_1_ –T_2_ difference is, say, 4 months apart)?

The study of a ‘sustained effect’ of an educational or a psychological variable requires the use of longitudinal data–for example, say, the positive effect of T_1_ self-efficacy on T_2_ perceived feeling of energy, and the positive effect of T_2_ perceived feeling of energy on T_3_ academic performance. Importantly, from this example, a *sustained effect* of T_1_ self-efficacy on T_2_ perceived feeling of energy (i.e., T_1_ self-efficacy → T_2_ energy) would require the use of a longitudinal methodological design, either experimentally, non-experimentally, or a combination of both (e.g., the experimental manipulation of self-efficacy at T_1_ to determine its sustained effect on subsequent variables). An examination of the Educational Psychology literature shows that in recent years, a number of researchers have used different longitudinal designs (e.g., a multi-wave panel design) to identify sustained effects of psychological variables [11, 65, 67–69]. In one of our earlier longitudinal studies that involved the measurement of different psychological and achievement-related constructs at different time points, for example, we found that self-efficacy at T_3_ positively predicted academic achievement at T_5_ (β = .23, *p* < .001) [65]. In a similar vein, Liem and his colleagues [12] reported an interesting line of evidence, which supported the use of an autoregressive longitudinal design (e.g., T_1_ personal best goals → T_1_ academic flow, β = .74, *p* < .05, and T_2_ personal best goals → T_2_ academic flow, β = .59, *p* < .05).

Despite the significance of longitudinal research designs in social sciences [12, 70–72], we note that opportunities for such engagement are not always possible and/or feasible. Time constraint, logistic difficulties, limited financial resources, etc. make it somewhat impossible at times for researchers to collect data on more than one occasion. Our recent research development [1, 7, 16] has provided some interesting methodological insights for consideration. One unique methodological insight, we contend, considers the development of a distinct entity, or construct (e.g., the development of a ‘sustaining’ entity), that could act as a proxy measurement for the ‘sustained effect’ of a psychological variable (e.g., T_1_ self-efficacy → T_2_ perceived feeling of energy). This proposed concept of a ‘sustaining’ concept, or entity, may allow us to delve into a person’s testament of desire and/or intent to continue on with his/her course of action without the use of longitudinal data (e.g., “I want to continue on and do not want to stop with what I am doing (e.g., learning a particular task in Algebra)”).

Inclusion of a ‘sustaining’ concept, or a sustaining entity, may address and accommodate the major shortcoming of a researcher’s inability to engage in longitudinal data collection for the purpose of statistical analysis of predictive flows and/or sustained effects of psychological and achievement-related constructs. In the context of schooling then, where opportunities for longitudinal research undertakings are limited, a compensatory measure may consist of the following equivalency (Note: we denote the concept of equivalency with the notation of ‘≈‘):

$$T_{1}\;\mathrm{Self}‐\mathrm{efficacy}\to T_{2}L_{2}\approx \mathrm{Self}‐\mathrm{efficacy}\to \mathrm{Sustaining}\to L_{2}$$

From the above mentioning, where longitudinal data are not available, a researcher may wish to measure and assess the impact of T_1_ self-efficacy on T_2_ optimal best (i.e., T_1_ Self-efficacy → T_2_ L_2_), for example, with the creative use of the aforementioned proposed concept (i.e., Self-efficacy → Sustaining concept → L_2_). Upon inspection, of course, we acknowledge that the above equivalency is not completely exact and/or accurate. It is erroneous to conclude that longitudinal data and, likewise, cross-sectional data, using the sustaining concept would both yield identical, or almost identical, results. Having said this, however, we contend that it is innovative to consider a ‘substitution’ in the form of a distinct entity for usage, which could add theoretical and/or methodological contribution to the study of ‘sustained effects’, or temporally-displaced effects [65, 67], of educational and psychological constructs. Interestingly, in accordance with our proposition, a positive effect arising from the concept of sustaining could, in this case, serve as a ‘proxy’ indication of a sustained effect, or a temporally-displaced effect, of an educational or a psychological variable. For example, from the above mentioning, a positive effect of the sustaining concept on L_2_ (i.e., sustaining → L_2_, +ve β value) and, by the same token, a positive effect of self-efficacy on the sustaining concept (i.e., self-efficacy → sustaining, +ve β value) would indicate self-efficacy for academic learning is ‘sustained’ over time (i.e., the ‘sustained’ effect of self-efficacy on L_2_).

In summary, the proposition of ‘sustaining’, as a distinct construct, is innovative and may, in fact, assist with our focus of inquiry into the operational nature of a person’s perceived feeling of energy. One notable line of our research, as depicted in Fig 2, concerns the extent to which a person’s perceived feeling of energy would sustain his/her academic engagement (e.g., perceived feeling of energy → sustaining → L_2_, +ve β value). We rationalize that the effect of a perceived feeling of energy is not instantaneous but rather sustains, which then would yield a number of positive consequences for consideration. Subsidiary to this examination, we also seek to advance theoretical understanding into the sustained effects of both self-efficacy [9, 51, 54, 55] and personal resolve [6, 8, 21]–for example, the use of *structural equation modeling* (SEM) techniques [13, 73] could clarify and provide theoretical insights into comparative pathways or relationships (e.g., self-efficacy → personal resolve → sustaining → L_2_).

### The present study

The present study seeks to advance the study of optimal best [1, 2, 63] and optimization [7, 8, 16], via means of statistical testing of a structural model, which is shown in Fig 2. Our hypothesized model, significantly, reflects the operational nature of the psychological process of optimization [1, 7, 16]–for example, the activation and predictive nature of psychological variables, individually or in tandem with each other, may encourage the striving of achievement of optimal best. We rationalize that our inquiry, seminal at this stage, could add empirical and/or theoretical contributions into the ‘optimization’ of a student’s state of cognitive functioning [6–8] by means of an important psychological mechanism, namely, his/her perceived feeling of energy. Influenced by self-efficacy for academic learning [9, 51], a perceived feeling of energy could enhance a student’s state of personal resolve [6, 8, 21] and, in turn, individually or in tandem with each other, account for an improved state of cognitive functioning. Moreover, in terms of methodological significance, we propose an interesting concept for inclusion, which we rationalize could accommodate our inability to collect longitudinal data.

## Methods

### Sample and procedure

Six hundred and twenty-seven undergraduate students (N = 438 women, 189 men) from two universities in Taiwan participated in this study. The dataset forms part of our larger research project, which involves both secondary and university students from Australia, Malaysia, and Taiwan. Studies involving human participants are/were reviewed and approved by the University of New England Research Ethics Committee. We verbally sought permission and informed any participant who did not wish to take part to let us know at the onset of data collection. Written informed consent for participation was not required for this study given that all of the participants were adults (i.e., over the age of 18). This method of verbally seeking participatory consent, used by us on previous research undertakings [60, 63], is more convenient logistically and was approved by our university (Research Ethics Committee, Approval Number: HE13-025).

Our sampling was convenient (i.e., the use of only two universities when, in fact, there are more than 150 universities and colleges in Taiwan) as it is/was somewhat difficult nowadays to recruit university students as participants. In fact, in the area of Education, many scholars would concur and attest to the extreme difficulty of being able to recruit participants to consent to experimental and/or non-experimental studies. There are a number of factors that may account for this perceived difficulty, for example: curriculum and time constraint, financial resource limitation, institution’s (as a whole) and/or student’s unwillingness to take part in the study, etc. By the same token, we were also faced with limited financial and human resources, which made it logistically difficult to expand our dataset to the wider population in Taiwan.

The participants voluntarily took part in the study, knowing that they would not receive any incentive. The third and fifth author of this article coordinated and managed the data collection process, which involved administration of the questionnaires, data entry, and record keeping (i.e., the third author coordinated and managed the data collection process in University A, and the fifth author coordinated and managed the data collection process in University B). Five of the seven authors of this article (i.e., exception of the first and second author) assisted in the administration of the questionnaires, whereas four postgraduate students assisted with data entry into SPSS and Excel databases. The dataset was collected during the last week of October, 2019 and took approximately two weeks to complete (i.e., last week in October with one of the three universities, and first week in November with the other two universities). The participants were briefed early in October, 2019 that a study was undertaken and that a survey would be administered in late October.

Despite the popularity of online approaches to data collection, we chose to use the traditional face-to-face, hard-copy methodological as this would ensure a better response rate. Likert-scale measures, which took approximately 30 minutes to complete, were administered by the postgraduate students in both lectures and tutorial classes. Participants were given five minutes at the end of the data collection process to ask questions, seek clarification, etc. A few postgraduate students, likewise, assisted in the data entry of the Likert-scale responses, using SPSS and Excel databases. The SPSS and Excel databases were eventually merged into one SPSS database for statistical analyses. Overall, all the participants were full-time students, enrolling in Liberal Arts, Engineering and Sciences, and Nursing/Medicine degree programs.

### Instruments

Overall, from Fig 2, there are six distinct variables with corresponding Likert-scale measures with ratings ranging from 1 to 5 –namely: 1 (Complete Not True), 2 (Not True), 3 (Neutral), 4 (True), and 5 (Completely True). Reliability estimates for the six subscales are shown in Table 1. In the next section of the article, we discussed our statistical analyses for the factorial structures of energy and sustaining concepts.

**Table 1:** Correlations between mean scale scores.

													Total		Female		Male	
	1		2		3		4		5		6	Rel	Mn	Std.	Mn	Std.	Mn	Std.
▪ *Self*	1.00											.79	3.75	0.56	3.81	0.54	3.63	0.60
▪ *Energy*	.58	**	1.00									.73	3.70	0.60	3.76	0.57	3.58	0.64
▪ *Current*	.42	**	.35	**	1.00							.82	3.94	0.65	4.01	0.63	3.78	0.66
▪ *Resolve*	.57	**	.56	**	.43	**	1.00					.86	3.92	0.59	3.97	0.58	3.81	0.58
▪ *Sustain*	.46	**	.47	**	.31	**	.40	**	1.00			.76	3.79	0.66	3.85	0.62	3.63	0.72
▪ *Optimal*	.52	**	.55	**	.48	**	.64	**	.47	**	1.00	.81	3.79	0.61	3.86	0.58	3.64	0.63

Note

**. Correlation is significant at the 0.01 level (2-tailed). Rel = reliability, Mn = mean, Std. = standard deviation.

1. **Personal resolve.** We adapted five items from recent research development [e.g., 6, 46] to measure and assess the psychological concept of personal resolve, which include, for example: “I will do whatever it takes to master my academic studies at university” and “I have a strong desire to succeed in my academic studies at university”.
2. **Personal belief of efficacy for learning.** We adapted five items from the Motivated Strategies for Learning Questionnaire [74, 75] to measure and assess the concept of personal belief of efficacy for academic learning [9, 51]. The items included, for example: “I believe I will receive excellent grades in classes at this university” and “I expect to do well academically in my classes for different subjects (e.g., Psychology)”.
3. **Current best practice, L**_**1**_. From theorization of best practice [1, 2], we recently developed two subscales to measure and assess both L_**1**_ and L_**2**_. Each original scale, developed in 2016 [2] consisted of eight items. Our subsequent empirical research [e.g., 6, 28, 60] led us to refine the two original subscales (i.e., psychometric properties), each consisting of five items–for example: “I am content with what I have accomplished so far at this university” and “I can achieve what is being asked of me at this university”.
4. **Optimal best practice, L**_**2**_. Similar to current best practice, L_**1**_, our revision of the original subscale of optimal best practice (i.e., psychometric properties led us to refine the subscale) [e.g., 6, 28, 60], L_**2**_, resulted in five items for usage–for example: “I can achieve much more at university than I have indicated through my work so far” and “I want to learn and do more at university”.
5. **Energy.** We considered some of the keywords mentioned previously from our students (e.g., stamina) to develop the Personal Energy Subscale, which has four items–for example: “I feel ‘alive’ whenever I think of my academic studies (e.g., mathematics)” and “I have this state of ‘adrenaline’ whenever I think of my academic studies (e.g., mathematics)”.
6. **Sustaining.** Our mentioned Sustaining Subscale has four items, which measure and assess the concept of sustaining–for example: “I want to continue on and do not want to stop with what I am doing (e.g., learning)” and “I want to prolong this (e.g., my learning) as much as possible”.

### Data analyses

Our data analyses, overall, consisted of two main stages: (i) a factorial analysis [13, 76] to explore the nature of the concepts of energy and sustaining, and (ii) a complete structural model to validate our original *a priori* model (Fig 2), using structural equation modelling (SEM) techniques [13, 76]. Existing research development has, to date, found sound and consistent psychometric properties for the two levels of best practice subscales, as well as the self-efficacy subscale. We appreciate and highlight that structural equation modeling (SEM) is advantageous when compared to other multivariate techniques, as it enables researchers to explore both direct and indirect effects, as well as to test for potential mediating effects for further development (e.g., experimental undertaking). The provision of various goodness-of-fit index values, likewise, allows researchers to test and compare competing *a priori* and *a posteriori* models [73, 77]. As such, the goodness-of-fit index values and the subsequent acceptance of an *a posteriori* model and the rejection of an *a priori* model, for example, would assist in confirmation of an original hypothesized model. We start off by exploring the factorial structures of energy and sustaining.

### Confirmatory factor analysis

Of the six theoretical concepts under investigation, energy and sustaining are relatively new in terms of conceptualization and empirical undertaking–on this basis, it is of interest for us to explore the following:

- *Perceived Feeling of Energy*: A one-factorial structure of energy in which we free the factor loadings of the four individual items, denoted as X_1_, X_2_, X_3_, and X_4_, onto a latent factor, denoted as ξ_1_, titled ‘Energy’ (i.e., to represent our proposed concept of ‘a perceived feeling of energy’).
- *Sustaining*: A one-factorial structure of sustaining in which we free the factor loadings of the four individual items, denoted as X_1_, X_2_, X_3_, and X_4_, onto a latent factor, denoted as ξ_1_, titled ‘Sustaining’ (i.e., to represent our proposed concept of ‘a sustained effect’).

We used the statistical software package M*Plus 8*.*5* [78] to assist with analyses of the two one-factor models. Likewise, SPSS 25 was used for descriptive statistics (e.g., data screening). Per guidance as indicated from existing research [13, 76], we performed an initial data screening test to ensure multivariate normality and the justification of using maximum likelihood (ML) estimates to test our hypothesized model (e.g., kurtosis values ranging from -.34 to .41 (Std error = .20) and skewness values ranging from -.44 to -.10 (Std error = .10) for energy; kurtosis values ranging from -.24 to .11 (Std error = .20) and skewness values ranging from -.43 to -.03 (Std error = .10) for sustaining. From previous research undertakings [12, 79], we considered the following indices to assist us with the gauging of appropriate model fits: the *Comparative Fit Index* (CFI)(i.e., CFI value > .95), the *Tucker Lewis Index* (TLI)(i.e., TLI value > .95) the *Root Mean Square Error of Approximation* (RMSEA)(i.e., RMSEA value < .07), the χ^2^ test statistic, and an evaluation of parameter estimates [80].

Prior to our confirmatory factor analysis undertakings, we performed an initial exploratory factor analysis (EFA) [81, 82] for the Energy Subscale and the Sustaining Subscale. Due to the limitation of space and our emphasis on confirmatory factor analysis, which is more robust and is of a priority, we have not devoted too much discussion here. However, our initial exploratory factorial analyses (e.g., Kaiser-Meyer-Oklin value = .723, Bartlett’s Test of Sphericity, *p* < .05 for energy, and Kaiser-Meyer-Oklin value = .766, Bartlett’s Test of Sphericity, *p* < .05 for sustaining) showed the presence of a one component (e.g., eigenvalue exceeding 1, explaining 56.42% of the variance for energy, and eigenvalue exceeding 1, explaining 59.09% of the variance for sustaining). Our subsequent confirmatory factor analyses, likewise, supported the exploratory factorial findings, confirming a one-factorial structure for both subscales. A one-factorial structure for the Energy Subscale is evident, as indicated by the various goodness-of-fit index values: χ^2^/dƒ = 9.49, CFI = .97, and TLI = .91, RMSEA = .12 (Li90 = .073, Hi90 = .167). Modification fit indices suggested the freeing of a correlation between two errors for Item 2 and Item 3, resulting in an *a posteriori* analysis. The analysis of the *a posteriori* model improved the model fit, as supported by the following index values: χ^2^/dƒ = 2.35, CFI = .99, and TLI = .98, RMSEA = .046 (Li90 = .001, Hi90 = .127). The Δχ^2^ test likewise showed statistical significance between the *a priori* model and the *a posteriori* model: 16.65, *p* < .001. On this basis, we accepted the *a posteriori* model, which showed factor loadings that ranged from .57 (λ_X41_) to .65 (λ_X11_), *p* < .001. A one-factorial structure for the Sustaining Subscale, similarly, indicated a good model fit: χ^2^/dƒ = 6.59, CFI = .98, and TLI = .93, RMSEA = .095 (Li90 = .051, Hi90 = .146). The factor loadings ranged from .51 (λ_X41_) to .79 (λ_X21_), *p* < .001.

### Structural equation modelling

Correlations between mean scale scores are shown in Table 1. The results established from the factorial structure analyses substantiated our SEM undertakings, which consisted of a baseline model. Overall, from the hypothesized *a priori* model, there are six latent factors: self-efficacy (5 items), energy (4 items), L_1_ (5 items), personal resolve (5 items), sustaining (4 items), and L_2_ (5 items). The baseline model, Model M_0_, as indicated in Fig 2, showed the freeing of the following structural paths: Path A_1_ (self-efficacy → energy), Path B_1_ (energy → L_1_), Path B_2_ (energy → L_2_), Path B_3_ (energy → personal resolve), Path C_1_ (L_1_ → personal resolve), Path C_2_ (L_1_ → sustaining), Path C_3_ (L_1_ → L_3_), Path D_1_ (personal resolve → sustaining), and Path E_1_ (sustaining → L_2_). The results of this base-line model, using covariance matrices as correlation matrix analysis is known to have potential problems (e.g., producing incorrect goodness-of- fit index values) [77, 83] are relatively modest in terms of fit–for example: χ^2^/dƒ = 2.92, CFI = .91, TLI = .90, RMSEA = .055 (Lo90 = .051, Hi90 = .059).

Model M_0_ is somewhat restricted and does not allow us to gauge into potential mediating mechanisms of energy, L_1_, personal resolve, and sustaining between self-efficacy and L_2_. In order to address this issue, and based on Baron and Kenny’s [84] recommendation, we freed five additional paths (Note: depicted as dotted lines in Fig 2): Path A_2_ (self-efficacy → personal resolve), Path A_3_ (self-efficacy → sustaining), Path B_4_ (energy → sustaining), Path D_2_ (personal resolve → L_2_), and Path E_1_ (sustaining → L_2_). The respecification of a base-line model and, subsequently, a comparison of two competing models (e.g., Model M_0_ *versus* Model M_1_) require the use of the Δχ^2^ test, as well as an inspection of the goodness-of-fit index values. The *principle of parsimony* [13, 14], in this case, commends the acceptance of a less restricted model. The results of the respecified model, Model M_1_, showed an improvement in model fit: χ^2^/dƒ = 2.54, CFI = .92, TLI = .91, RMSEA = .050 (Lo90 = .046, Hi90 = .053), and Δχ^2^_(Δdƒ = 5)_ = 102.81, *p* < .001.

**Fig 3:**
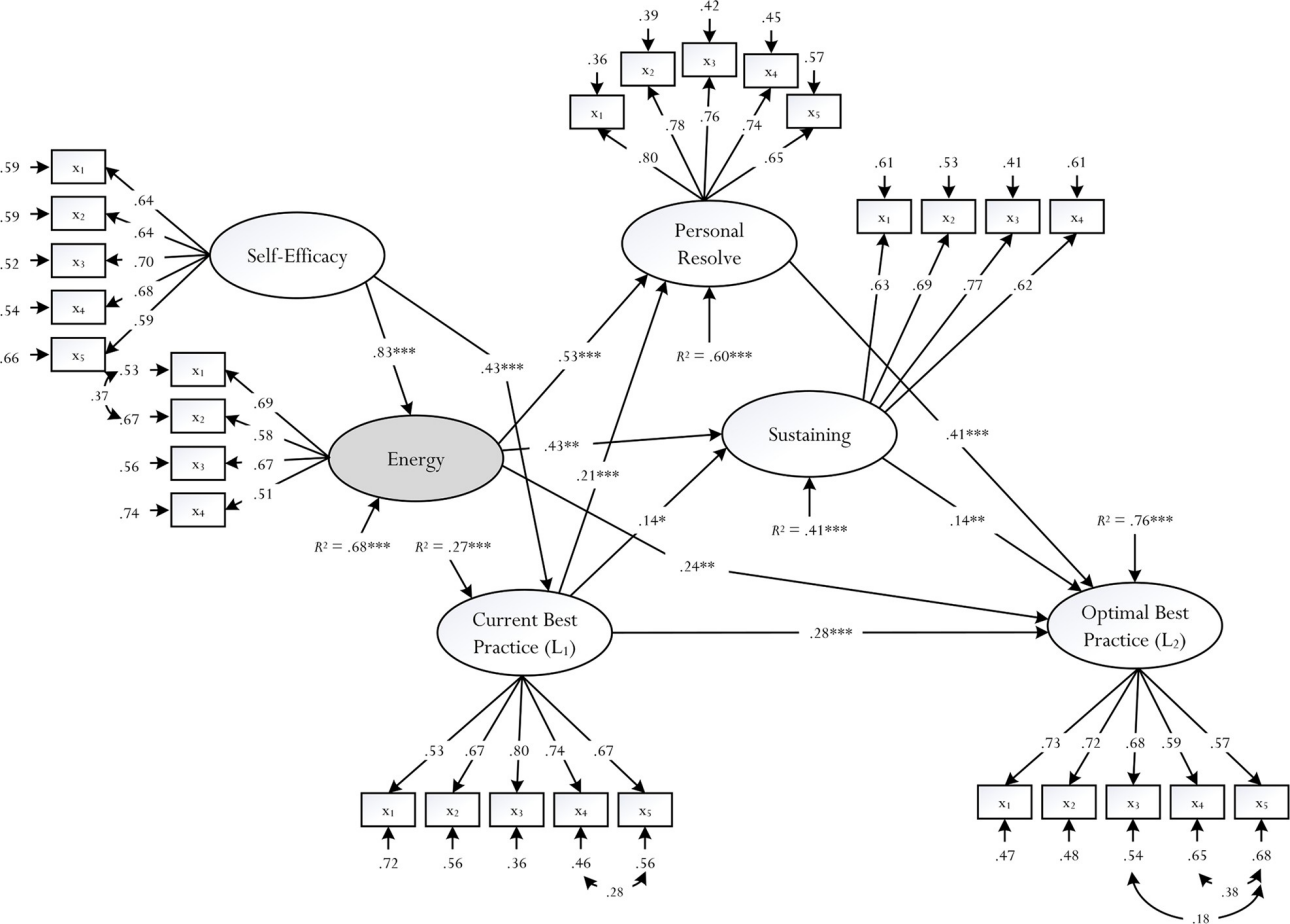
Final solution. Statistically significant paths at * *p* < .05, ** *p* < .01, *** *p* < .001 are shown, whereas non-statistically significant paths have been omitted for clarity. Measured indicators for each proposed latent factor (e.g., self-efficacy) are depicted as X_**1**_, X_**2**_, …, X_**n**_, where n = 1, 2, …, 5.

Model M_1_, although not optimal in nature [13, 73], is somewhat sound for further discussion and future research development. Fig 3 visually depicts the final solution of Model M_1_ for discussion. Further analyses by which we decomposed the total effects, as well as to stipulate mediating effects are shown in Tables 2 and 3. An inspection of Fig 3 is interesting as it shows that our original hypothesized model was partially supported–for example: (i) the role of self-efficacy as an antecedent of a perceived feeling of energy (β = .83, *p* < .001) and L_1_ (β = .43, *p* < .001), (ii) the positive effect of a perceived feeling of energy on personal resolve (β = .53, *p* < .001), sustaining (β = .43, *p* < .01), and L_2_ (β = .24, *p* < .01), (iii) the positive effect of L_1_ on personal resolve (β = .21, *p* < .001), sustaining (β = .14, *p* < .05), and L_2_ (β = .28, *p* < .001), (iv) the positive effect of personal resolve on L_2_ (β = .41, *p* < .001), and (v) the effect of sustaining on L_2_ (β = .14, *p* < .01).

**Table 2:** Decomposition of direct + indirect effects.

	Direct	*p*	Indirect	p	Total	*p*
On Optimal Best Practice						
▪ Of Sustain	.14	**	-		.14	**
▪ Of Personal Resolve	.41	***	-.01		.40	***
▪ Of Current Best Practice	.28	***	.11	***	.39	***
▪ Of Energy	.24	**	.31	***	.55	***
▪ Of Self-Efficacy	-		.70	***	.70	***
On Sustain						
▪ Of Personal Resolve	-.06		-		-.06	
▪ Of Current Best Practice	.14	*	-.01		.13	*
▪ Of Energy	.43	**	-.02		.41	**
▪ Of Self-Efficacy	.20		.39	***	.59	***
On Personal Resolve						
▪ Of Current Best Practice	.21	***	-		.21	***
▪ Of Energy	.53	***	.02		.55	***
▪ Of Self-Efficacy	.13		.55	***	.68	***
On Current Best Practice						
▪ Of Energy	.10		-		.10	
▪ Of Self-Efficacy	.43	***	.09		.52	***
On Energy						
▪ Of Self-Efficacy	.83	***	-		.83	***

Note

* *p* < .05

** *p* < .01

*** *p* < .001.

**Table 3:** Decomposition of indirect effects and potential mediating effects.

Predictor	Mediator				Outcome	Β	*p*
Self-Efficacy	Energy				Personal Resolve	.457	***
▪ Self-Efficacy	Energy				Personal Resolve	.438	***
▪ Self-Efficacy	Energy		Current Best Practice		Personal Resolve	.018	
Self-Efficacy	Energy				Sustain	.336	**
▪ Self-Efficacy	Energy				Sustain	.351	**
▪ Self-Efficacy	Energy		Current Best Practice		Sustain	.012	
▪ Self-Efficacy	Energy		Personal Resolve		Sustain	-.027	
▪ Self-Efficacy	Energy	Current Best Practice		Personal Resolve	Sustain	-.001	
Energy	Sustain				Optimal Best Practice	.056	*
▪ Energy	Sustain				Optimal Best Practice	.059	*
▪ Energy	Current Best Practice		Sustain		Optimal Best Practice	.002	
▪ Energy	Personal Resolve		Sustain		Optimal Best Practice	-.005	
Self-Efficacy	Sustain				Optimal Best Practice	.081	**
▪ Self-Efficacy	Sustain				Optimal Best Practice	.028	
▪ Self-Efficacy	Energy		Sustain		Optimal Best Practice	.049	*
▪ Self-Efficacy	Current Best Practice		Sustain		Optimal Best Practice	.009	
▪ Self-Efficacy	Personal Resolve		Sustain		Optimal Best Practice	-.001	
▪ Self-Efficacy	Energy	Current Best Practice		Sustain	Optimal Best Practice	.002	
▪ Self-Efficacy	Energy	Personal Resolve		Sustain	Optimal Best Practice	-.004	
▪ Self-Efficacy	Current Best Practice	Personal Resolve		Sustain	Optimal Best Practice	-.001	
Current Best Practice	Personal Resolve				Optimal Best Practice	.085	***
▪ Current Best Practice	Personal Resolve				Optimal Best Practice	.086	***
▪ Current Best Practice	Personal Resolve		Sustain		Optimal Best Practice	-.002	
Energy	Personal Resolve				Optimal Best Practice	.219	***
▪ Energy	Personal Resolve				Optimal Best Practice	.215	***
▪ Energy	Personal Resolve		Sustain		Optimal Best Practice	-.005	
▪ Energy	Current Best Practice		Personal Resolve		Optimal Best Practice	.009	
Self-Efficacy	Personal Resolve				Optimal Best Practice	.27	***
▪ Self-Efficacy	Personal Resolve				Optimal Best Practice	.054	
▪ Self-Efficacy	Personal Resolve		Sustain		Optimal Best Practice	-.001	
▪ Self-Efficacy	Energy		Personal Resolve		Optimal Best Practice	.177	***
▪ Self-Efficacy	Energy	Personal Resolve		Sustain	Optimal Best Practice	-.004	
▪ Self-Efficacy	Current Best Practice		Personal Resolve		Optimal Best Practice	.037	*
▪ Self-Efficacy	Current Best Practice	Personal Resolve		Sustain	Optimal Best Practice	-.001	
▪ Self-Efficacy	Energy	Current Best Practice		Personal Resolve	Optimal Best Practice	.007	
Self-Efficacy	Energy				Optimal Best Practice	.45	***
▪ Self-Efficacy	Energy				Optimal Best Practice	.194	**
▪ Self-Efficacy	Energy		Current Best Practice		Optimal Best Practice	.024	
▪ Self-Efficacy	Energy		Personal Resolve		Optimal Best Practice	.177	***
▪ Self-Efficacy	Energy		Sustain		Optimal Best Practice	.049	*
▪ Self-Efficacy	Energy	Current Best Practice		Personal Resolve	Optimal Best Practice	.007	
▪ Self-Efficacy	Energy	Current Best Practice		Sustain	Optimal Best Practice	.002	
▪ Self-Efficacy	Energy	Personal Resolve		Sustain	Optimal Best Practice	-.004	

Note

* *p* < .05

** *p* < .01

*** *p* < .001. For the simplicity of space, we have omitted non-statistically significant relationships.

An inspection of Fig 3 and from Tables 2 and 3, we note some interesting pathways or trajectories, which substantiated our original proposition into the accomplishment of L_2_ –for example: self-efficacy → energy → sustaining → L_2_ (β = .049, *p* < .05); self-efficacy → energy → personal resolve → L_2_ (β = .177, *p* < .001); and self-efficacy → L_1_ → personal resolve → L_2_ (β = .037, *p* < .05). One notable pathway (i.e., self-efficacy → energy → sustaining → L_2_), in this case, emphasized the potent effect of sustaining, whereas another pathway (i.e., self-efficacy → energy → personal resolve → L_2_) substantiated the central role of personal resolve.

## Discussion of results

The present study, non-experimental in nature, is innovative for its attempt to explore the operational nature of a psychological concept, which we termed as a ‘perceived feeling of energy’. The underlying premise of our conceptualization concerned the extent to which a perceived feeling of energy would act a motivational driver to assist with and/or to account for the process of optimization [7, 32, 33]. That a perceived feeling of energy, shaped by self-efficacy for academic learning [9, 51], could potentially motivate a student to strive for achievement of optimal best in an academic subject matter. Importantly, of course, we rationalized that a perceived feeling of energy could ‘optimize’ a person’s state of functioning from T_1_ to T_2_. To this end, complementing our focus of examination, we inquired into the supposition that a perceived feeling of energy would also have a sustained effect on different types of adaptive outcomes (e.g., personal resolve). To consider this possibility, we creatively conceptualized a corresponding entity, which we firmly believe could compensate for a researcher’s shortcoming of having the availability of longitudinal data for analysis.

The results of our inquiry, overall, have advanced theoretical understanding into the ‘optimization’ of a person’s state of cognitive functioning. An improved state of cognitive functioning, denoted as L_2_, is positive, proactive, and motivational, reflecting some ‘form’ of optimization [7, 33]. By all account, this consideration into the notion of optimization of an internal state of functioning [7, 16, 33] is extremely insightful but somewhat difficult to measure, assess, and validate. The act, or mechanism, of an ‘optimizing effect’, denoted as being some unknown entity [7] (i.e., not necessary a β value), which may account for a person’s achievement of L_2_, is unexplained at this stage. Our established evidence, to a certain degree, has elucidated this complexity by confirming specific pathways or trajectories that could help explain the optimization of a person’s optimal best. Rather unique, of course, is the finding that we obtained, which showed the ‘operational nature’ of energy, or a perceived feeling of energy.

### Theoretical contributions to the study of optimization

Advancing the important line of inquiry into the achievement of optimal best [1, 2, 12], we proposed a psychological concept known as a ‘perceived feeling of energy’ for examination. We rationalized that a perceived feeling of energy, as a central ‘optimizing driver’, would help facilitate a student’s state of cognitive functioning. This inquiry into the validation of a perceived feeling of energy would, significantly, provide additional theoretical insights into the study of human optimization [1, 7, 16]. Human optimization, according to some researchers [1, 2, 7, 16], is an underlying psychological process that may explain how one achieves an optimal level of best practice from his/her current level of best practice (i.e., L_1_ → L_2_). Phan and his colleagues [7] recently considered the potentiality for a person’s ‘experience of energy’ to account for and/or to play some ‘optimizing role’ in the optimization of L_1_, which then would result in the achievement of L_2_.

Overall, we postulated that a perceived feeling of energy, as a positive and motivational entity [7, 16], would directly account for a person’s achievement of optimal best (i.e., a perceived feeling of energy → L_2_) and mediate an identified source of information (i.e., self-efficacy for academic learning) onto other types of adaptive outcomes (e.g., self-efficacy → a perceived feeling of energy → personal resolve). We reason that positive pathways, or positive trajectories, would largely, but not solely, substantiate the recent theorization into the optimization of a person’s achievement of optimal best. The solution shown in Fig 3 and Tables 2 and 3 consisted of the following:

■ The role of personal self-efficacy for academic learning as an antecedent, or source of information, in the prediction of L_1_ and a perceived feeling of energy.
■ A perceived feeling of energy served as a potent predictor of personal resolve, sustaining, and L_2_.
■ Personal resolve and sustaining served as predictors of L_2_.
■ The validation of the concept of sustaining, which could affirm the sustained effects of, say, L_1_ and a perceived feeling of energy.
■ Comparative pathways, or trajectories, of accomplishment of L_2_, which emphasize the importance of a perceived feeling of energy, personal resolve, and sustaining (e.g., self-efficacy → energy → sustaining → L_2_).

The aforementioned summation is insightful and may help advance the study of human optimization [1, 7, 16]. Theoretical and empirical inquiries into the process of optimization place emphasis on one common theme: how do we ‘optimize’ a person’s state of functioning? The coining of the term ‘optimization’ in the area of academic performance and personal well-being by Fraillon [1], interestingly, is somewhat of a conundrum as, to date, there is no solid empirical evidence for support. Phan and his colleagues [7] subsequently provided a theoretical framework, which the authors believed would explain the intricacy of the process of optimization. One notable distinction of this theoretical account relates to the inclusion of a psychological concept known as ‘energy’ [7, 16, 47]. According to the authors’ theorization, energy could act as a central driver to help optimize a person’s state of functioning from T_1_ to T_2_. The complexity of the theorization of optimization [6, 8, 16], however, makes this process somewhat difficult to measure, assess, and/or validate. For example, considering recent research development [7], how would we accurately measure the intricacy of a person’s experience of energization? In a similar vein, how would we measure and determine the ‘complete optimization’ of a student’s improved state of cognitive functioning from T_1_ to T_2_ in a subject matter?

Our theoretical contribution, significantly, consisted of a preliminary validation of an optimizing concept, which we termed as perceived feeling of energy. Interestingly, we noted that self-efficacy served as an important source of information in the prediction of a perceived feeling of energy, confirming existing research [54, 55] into the operational nature of Bandura’s [9] social cognitive theory. Furthermore, correlational analysis of non-experimental data resulted in the establishment of evidence that confirmed the ‘sustained’ effect of a perceived feeling of energy (e.g., a perceived feeling of energy → personal resolve). By all account, of course, a predictive effect of a perceived feeling of energy, denoted as a β value, does not truly equate to the notion of ‘optimization’ and/or an ‘optimizing effect’. At best, from our point of view, there is empirical grounding to suggest that a perceived feeling of energy would serve as a sound determinant of future outcomes (e.g., L_2_). A lack of a perceived feeling of energy (e.g., “I do not feel alive while I am doing this …..”), in contrast, would demotivate and weaken a student’s personal resolve (i.e., a perceived feeling of energy → personal resolve, -ve β value), resulting in his/her sub-optimal learning experiences.

Our results also substantiated the relevance and applicability of personal resolve as a predictor and potential mediator of future outcomes. This finding (i.e., personal resolve → L_2_) coincides with evidence from previous studies [e.g., 6, 60], which emphasizes the importance of a student’s state of personal resolve. Decisiveness, mental resolute, and one’s unwavering focus to strive for success, regardless of difficulties, obstacles, hardships, etc. will form an important basis, helping to direct and motivate a person to achieve his/her optimal best. Interestingly, aside from optimal best practice, Phan and his colleagues’ recent studies found that personal resolve also positively influenced other psychological variables–for example: academic striving [60], academic liking experience [21], and improved effort expenditure [46]. On a wider scale, personal resolve may act as a source of grit [85], instilling and/or heightening a person’s cognitive fortitude to persist and strive for educational and/or non-educational success. For example, non-academically, we speculate whether personal resolve could propel a professional tennis player to overcome her ‘hardship’ (e.g., match fatigue) and win a perceived unwinnable match? Personal resolve, in this case, may initiate a heightened state of persistence, mental strength, and/or unwavered (or unwavering) elief in success, which then would result in her sense of determination and concentration to succeed.

### Practical implications for consideration

The significance of our research, as summarized in Fig 3, Tables 2 and 3, also lies in the recommendation of different types of educational and/or non-educational practice for consideration. One notable feat for promotion and cultivation entails a person’s aspiration and striving to achieve a level of optimal best in a specific domain of functioning–for example, within the context of academic learning, a secondary school student aspires to achieve a level of optimal best in Algebra, or a university student wishes to achieve a level of optimal best in her Honors studies in Psychology. Our findings reflect the important tenets of human optimization [7, 16] and positive psychology [4, 5, 27] and may, interestingly, provide grounding for the development of pedagogical strategies, educational programs, etc. for implementation. Foremost, of course, is the capitalization of the psychological concept of a perceived feeling of energy, which may serve to instill and optimize a strong sense of personal resolve, resulting in an improved state of cognitive functioning. On this basis, for the context of this article, we consider three distinct possibilities:

- The *use of verbal discourse*. Different types of *verbal discourse*, we contend, are effective sources of information, which may account for improvement in academic performance, personal resource, and self-beliefs (e.g., self-efficacy) [9, 86–88]. Timely feedbacks, involving attributional feedback (e.g., “You’re working very hard, Toan”: Effort feedback), positive feedback (e.g., “Bau-Yi, your assignment looks great! It’s clear that you take pride in your work and you take time to it superbly”), encouraging feedback (e.g., “You can do this difficult task, Thomas; I know you can.”), and guidance feedback (e.g., “This is how I want you to do this, Aaron; watch what I am doing and model yourself …..”) may help to facilitate self-confidence, positive emotions (e.g., situational happiness), and/or feel-good experiences. We acknowledge, however, that excessive use of verbal discourse could, in fact, lose potency, effectiveness, and/or credibility. It is logical, in this sense, for educators to consider the use of timely and well-paced feedbacks, of different types, which could help energize a student’s state of personal resolve and, in turn, motivate and optimize his/her learning experiences. For example, imparting positive feedback to a student may instill ‘feel-good’ and buoyant experiences, which could act as perceived sources of energy.
- The *importance of confidence building*. *Self-confidence*, a part of a person’s self-belief system, we contend, may improve, enhance, and/or facilitate a person’s engagement of different types of adaptive outcomes [32]. “I am extremely confident that I am able to….” is a personal testament that is motivational, optimistic, and buoyant, which in turn may act to stimulate and motivate a person’s progress. Moreover, from our point of view, self-confidence during the course of an action (e.g., a senior citizen’s engagement in learning of Buddhist meditation) may energize a person’s state of motivation (e.g., self-confidence, we contend, may strengthen a person’s persistence), resulting in his/her personal resolve to strive for optimal best. ‘Confidence building’, taking into consideration the importance of *social cognitive theory* [9, 10], may involve, for example, the use of vicarious learning (e.g., the importance of role modelling and, in particular, observation) and/or verbal discourse (e.g., the use of positive feedback and/or encouraging feedback), both of which effective sources of information. In a similar vein, personal training involving the development of self-regulatory skills (e.g., the teaching and training of evaluation and monitoring skills) [89–91] may also help to facilitate and/or strengthen a person’s state of self-confidence. For example, self-evaluating and monitoring one’s own progress and, consequently, reflection and repeated practice to accommodate and/or improve a course of action may strengthen his/her personal resolve and self-confidence.
- *The importance of mastery*. Mastery, as opposed to performance-based learning, is a perceived positive feat, which may reflect a person’s proactive engagement and deep, meaningful learning experience [92–95]. Moreover, mastery in a subject matter may coincide with a person’s perceived value and/or interest [96–99], resulting his/her intrinsic motivation and desire to achieve educational and/or non-educational success. For example, in one of the earlier studies, Senko and Miles [98] found that students pursuing a mastery goal reported more interest in the course material than those students who avoided this goal (p. 572). On this basis, a focus on the structuring of appropriate learning outcomes and/or subject contents may be warranted as this could, in effect, operate to energize and optimize a student’s learning experience. Subject contents that have authenticity and daily-life relevance (e.g., a particular content that can be applied to a real-life situation), for instance, are more likely to stimulate personal interest, intellectual curiosity, and intrinsic motivation, all of which could energize a student’s feel-good experience (e.g., making a student feels more ‘buoyant’).

### Methodological contributions and directions for consideration

One interesting contribution of our research undertaking relates to the importance of what is known as ‘methodological appropriateness’ [7]. Methodological appropriateness emphasizes the appropriateness of a methodological design that one could use to accurately investigate a theoretical concept and/or a relationship or relationships between different constructs. In this sense, aside from both theoretical and practical contributions, the present study has also shed some notable insights into the complex issues of methodology of measurement and assessment of the process of human optimization [6, 7, 16]. Established evidence, as summarized in the preceding sections (e.g., Fig 3), has led to the identification of three major caveats, which we discuss in this section of the article: the *nature of energy*, a *sustained effect of energy*, and the *process of optimization*.

#### 1. The nature of energy

What is energy, especially from a psychological perspective? Recent research development into the study of optimal best has incorporated a psychological concept known as ‘energy’ [7, 16, 47]. We contend, however, that the term energy is perplexing at best, especially from the perspective of its operational nature and measurement and assessment. How would we accurately measure and assess a person’s internal *state of energy*, which then would optimize and improve his/her functioning from T_1_ to T_2_ [7]? Interestingly, as we briefly described, one of our reviewers disagreed and commented on our use of the term ‘energy’. The reviewer suggested that energy, in this case, could simply be a characteristic of a person’s internal state of motivation. We appreciate this reviewer’s analytical critique as this collegial input, indeed, has potential ramifications, especially in relation to current development of the theory of human optimization [1, 7, 8] (e.g., a need, perhaps, for us to revise the theory of human optimization, which stipulates the importance of energy as a central driver [7]).

Indeed, aside from the analytical critique from one of our reviewers, we contend that validating the underlying nature of energy is somewhat complex and would require further conceptualization and/or methodological development. For example, incorporating the reviewer’s critique and subsequent suggestions, it is plausible that we could redefine and/or advance our scope in definition and coverage of the term ‘a perceived feeling of energy’. In a similar vein, of course, we argue that a 4-item questionnaire is somewhat limited in terms of capturing and/or illuminating the true complex nature of energy [7, 16]. Future research investigations, likewise, could consider expanding and developing additional items to complement our existing measure (i.e., 4 items). From our point of view, a non-experimental approach is somewhat limited in terms of scope and flexibility, which could shed interesting insights for consideration (e.g., the manipulation of self-efficacy for academic learning, which could affect a change in a person’s perceived feeling of energy). At best, we contend that a Likert-scale inventory, regardless of its coverage (e.g., 4 items *versus* 20 items), is more appropriate for the quantitative measurement and assessment of a person’s *perception* and/or *judgment* of his/her feeling of ‘being energized’ and not, in this case, the concept of energy itself.

Self-report measures are subjective and may yield unintentional or intentional bias, resulting in misjudgment and miscalibration of a person’s belief, feeling, experience, etc. [100, 101]. In this analysis, it would be enriching to consider other forms of assessment and measurement–for example, what do our observations of a person’s action and/or reaction to a particular context or situation tell us about his/her ‘state’ of energy? This consideration does not place emphasis on a person’s reporting of his/her perception of experience of energy; rather, the onus rests with the researcher to provide a sound and non-biased indication of observation of a person’s behavior, action, reaction, etc., which then would define and connote his/her internal state of energy. By the same token, of course, the psychological concept of energy is still in its early stage of research evolution. One such discourse may involve the conceptualization and development of an appropriate or an aligned methodological design that could soundly permit a researcher to accurately measure and assess the intricacy of energy, situated within the theory of human optimization [7, 16].

#### 2. A sustained effect of a perceived feeling of energy

The theory of human optimization [7, 16] describes one interesting aspect, which relates to the ‘sustaining’ of a psychological effect so that this could, indeed, help optimize a person’s state of functioning from T_1_ to T_2_ (Note: the duration of the T_1_ –T_2_ period may be 6 weeks, for example). As we discussed earlier, measuring, assessing, and determining a sustained effect of an educational or a psychological variable require some form of longitudinal examination, which may consist of data collected on multiple occasions–for example: a perceived feeling of energy in August, 2021, L_2_ in October, 2021, etc. One major pitfall that we acknowledge relates to our use of cross-sectional data, which made it extremely difficult to accurately confirm the notion of a sustained effect of a student’s perceived feeling energy.

**Fig 4:**
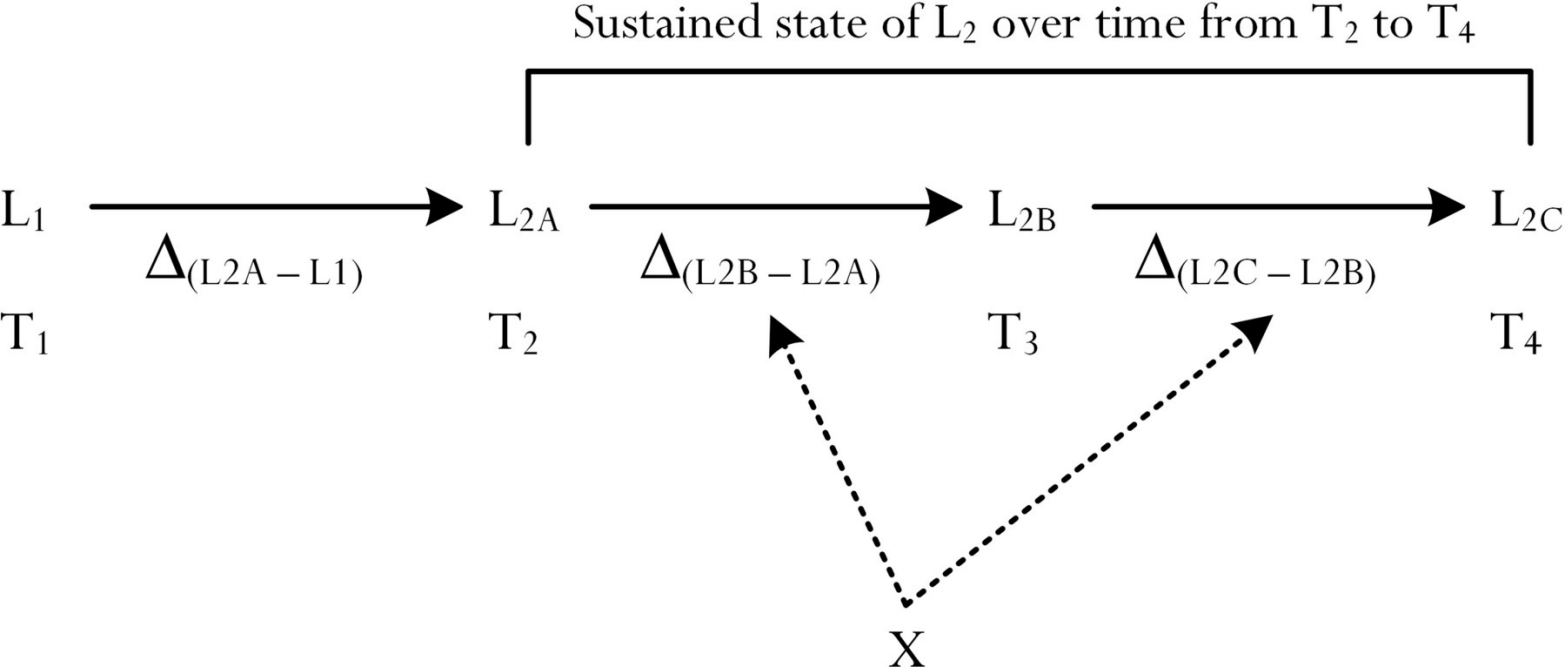
Conceptualization of measurement and assessment of L_2_. A conceptualization of a methodological design, which may consist of multiple time points: T_**1**_, T_**2**_,…, T_**n**_, where n = 1, 2,….. In this conceptualization for consideration, L_**1**_ is measured and assessed at T_**1**_ whereas L_**2**_ is measured and assessed at T_**2**_ (i.e., L_**2A**_) and beyond. A methodological design for usage is more explanatory when it encompasses both non-experimental (e.g., the use of a Likert measure at, say, T_**1**_) and experimental (e.g., an intervention, denoted as ‘X’, in between T_**2**_ and T_**3**_ or at T_**3**_).

As an in-depth analysis, consider Fig 4, which depicts a conceptualization for future research development. The notion of a sustained state of flow (i.e., the continuation of a student’s experience of L_2_) would require us to measure and assess L_2_ from a particular time point (e.g., March, 2021) and into the future (e.g., September, 2021). In a similar vein, the measurement of a sustained effect of a perceived feeling of energy on L_2_ (i.e., perceived feeling of energy → L_2_) would require us to consider the following methodological design (see Fig 4): the measurement of L_1_ at T_1_, the measurement of L_2_ at T_2_ (i.e., L_2A_), T_3_ (i.e., L_2B_), and T_4_ (i.e., L_2C_), and the interjection of a perceived feeling energy at T_2_ or at T_3_ (i.e., X). This stipulation, in turn, would allow us to measure and assess the sustained effect of a perceived feeling of energy at T_2_ on L_2_ at T_4_ (i.e., L_2C_). Statistically, using latent growth modelling (LGM) techniques [102, 103], we could identify the growth trajectory of L_2_ from T_2_ to T_4_. Moreover, a comparison in mean scores between two cohorts (i.e., Cohort A without any interjection and/or measure of a perceived feeling of energy *versus* Cohort B with an interjection) would allow us to explore and determine the potency of the interjection of ‘energy’ (e.g., energy could, in this case, improve and sustain a student’s L_2_)–for example, from Fig 4, we would expect to find Δ(L_2C_ –L_2B_) > Δ(L_2B_ –L_2A_) > Δ(L_2A_ –L_1_).

We acknowledge that nowadays, it is somewhat difficult for researchers to have flexible access to institutions, organizations, schools, etc. for the purpose of collecting longitudinal data. Time constraint, the availability of financial and/or human resources, participants’ willingness, etc. may limit opportunities for longitudinal research undertakings, resulting in imperfect and flawed methodological designs. As such, it is extremely difficult, if not improbable, for us to explore the proposed issue of a sustained effect of a perceived feeling of energy. Our inquiry into the operational nature of energy [7, 16, 47] resulted in the development of a related entity (i.e., the concept of ‘sustaining’), which we proposed could offer an alternative insight into the notion of a sustained effect of a variable. Despite this creativity, however, we argue that this distinct sustaining concept does not truly capture and/or encapsulate a sustained effect of an educational or a psychological variable. For example, in terms of statistical inference, what does the finding of self-efficacy → a perceived feeling of energy → sustaining → L_2_ (β = .049, *p* < .05) actually represent? How can we transform and/or equate this cross-sectional finding (i.e., β = .049, *p* < .05) of a ‘sustained effect’ with one that draws from the use of longitudinal data? In other words, is there any theoretical validity to infer that “self-efficacy → energy → sustaining → L_2_” (β = .049) is equivalent to, say, “T_1_ self-efficacy → T_4_ L_2_”?

#### 3. The process of optimization

The process of human optimization, as recently theorized [1, 7, 16], is intricate and requires sound and appropriate methodological designs for consideration. We acknowledge that, to date, we have used philosophical psychology [34], as a distinct research paradigm, to conceptualize new ideas and theoretical models for research development. We contend that theoretical models such *esoteric psychology* [38] and *holistic psychology* [25] are somewhat difficult to validate scientifically. This acknowledgement questions whether we have appropriate methodological designs, at this stage, to accurately measure and assess the notion of an ‘optimizing effect’ [7]. Self-report questionnaires, used in previous research studies [6, 28, 60, 63], are relatively effective for their non-intrusive nature and ability to gauge into associative patterns of psychological and achievement-related constructs (e.g., personal resolve → effort expenditure) [46]. Having said this, however, we recognize that self-report measures, situated within the context of non-experimental research are somewhat restricted, limiting us from seeking deep, meaningful understanding into the complexity of ‘internalized’ psychological experiences.

At present, we have no concrete evidence to substantiate the philosophical-derived model of optimization, which considers energy, or a perceived feeling of energy, as a central driver of human agency [7, 16, 47]. The notion of an optimizing effect and/or the totality of the underlying mechanism of optimization [7], as we acknowledged, is still ambiguous and requires further research development, especially in terms of methodological design and empirical validation. Phan and his colleagues [7] recently considered an interesting facet where the authors contended that a +ve β value, depicting a predictive effect of energy does not soundly and/or accurately depict the optimization of a person’s state of functioning. In other words, a structural model analysis that involves the use of non-experimental data, either cross-sectional or longitudinal, does not *truly* capture the full complexity and intricacy of the process of optimization. We appreciate that our findings and those established elsewhere [6, 28, 60] are somewhat limited as they simply detail *associative patterns* in relationship between psychological and achievement-related constructs (e.g., L_1_ → L_2_ *versus* personal resolve → L_2_). From this account, we advocate for the development of an alternative methodological design, or methodological designs, that could complement existing research methodologies (e.g., the use of non-experimental data) for usage. What researchers want to consider, in particular, is a proposition where a predictive effect, a mediating effect, and/or a moderating effect (e.g., β value) does not necessarily equate to an optimizing effect [7].

## Conclusion

The present research, coinciding with the study of positive psychology, addressed an interesting topical theme–namely, a person’s achievement of optimal best. Helping a person to experience and/or appreciate optimal best (e.g., a senior citizen experiencing optimal health after recovering from Covid-19) is an ambitious endeavor, which may involve the use and/or enactment of different pathways, means, opportunities, etc. Capitalizing on the tenets of positive psychology and, more importantly, our recent research development of the topic of human optimization, we proposed a psychological concept for examination–a perceived feeling of energy. A perceived feeling of energy, we argued, could act as a motivational ‘driver’ to propel a person’s course of action, resulting in improvement and/or progression from T_1_ to T_2_. Our results, drawn from structural equation modelling, are insightful, providing relevant information into the intricate nature of the proposed concept of energy. Innovatively, in the absence of longitudinal data, we constructed an additional construct that we believed would cast interesting insights into the ‘sustaining’ of a person’s optimal experience in a subject matter.

## Supporting information

S1 Appendix (DOCX)

S1 Table Covariance matrix. (PDF)
