# Supplementary material for: Empirical validation of the psychological concept of a perceived feeling of ‘energy’: Advancement into the study of positive psychology
Source: PLoS One. 2021 Nov 18;16(11):e0259762. doi: 10.1371/journal.pone.0259762 (PMC8601505; doi:10.1371/journal.pone.0259762)
Supplement: S1 Appendix — (DOCX) [file pone.0259762.s001.docx]

# Appendix

## Energy

1. I feel ‘alive’ whenever I think of my academic studies (e.g., mathematics).
2. I have this state of ‘adrenaline’ whenever I think of my academic studies (e.g., mathematics).
3. I feel that I have this ‘mental strength’, which could me succeed in academic studies (e.g., mathematics).
4. I have this internal ‘excitement’, which I feel is helping me in my academic studies (e.g., mathematics).

## Sustaining

1. I want to continue on and do not want to stop with what I am doing (e.g., mathematics learning).
2. I want to prolong this course of action (e.g., my mathematics learning) as much as possible.
3. I do not want to stop this course of action (e.g., my mathematics learning).
4. It is important for me to keep ‘sustaining’ with what I am doing (e.g., my mathematics learning).
