# Supplementary material for: Empirical validation of the psychological concept of a perceived feeling of ‘energy’: Advancement into the study of positive psychology
Source: PLoS One. 2021 Nov 18;16(11):e0259762. doi: 10.1371/journal.pone.0259762 (PMC8601505; doi:10.1371/journal.pone.0259762)
Supplement: S1 Table — (PDF) [file pone.0259762.s002.pdf]

Covariance Matrix from MPlus

Covariances

	MTL1	MTL5	MTL7	MTL9	MTL10
MTL1	0.472				
MTL5	0.238	0.595			
MTL7	0.232	0.275	0.612		
MTL9	0.198	0.251	0.318	0.609	
MTL10	0.205	0.219	0.235	0.288	0.660
EN1	0.183	0.292	0.216	0.225	0.180
EN4	0.190	0.198	0.240	0.180	0.152
EN5	0.196	0.184	0.244	0.217	0.186
EN6	0.145	0.176	0.183	0.145	0.133
CB4	0.117	0.099	0.099	0.094	0.192
CB5	0.128	0.104	0.125	0.137	0.196
CB6	0.144	0.155	0.161	0.173	0.209
CB7	0.153	0.124	0.145	0.174	0.217
CB8	0.167	0.145	0.164	0.164	0.217
RE1	0.196	0.207	0.242	0.224	0.227
RE2	0.164	0.165	0.156	0.168	0.186
RE3	0.178	0.182	0.221	0.205	0.166
RE4	0.152	0.158	0.170	0.194	0.151
RE7	0.172	0.195	0.206	0.223	0.156
SU1	0.183	0.119	0.201	0.248	0.141
SU5	0.184	0.159	0.141	0.172	0.167
SU6	0.210	0.148	0.148	0.169	0.158
SU7	0.194	0.137	0.180	0.182	0.186
OB2	0.181	0.172	0.206	0.222	0.234
OB4	0.186	0.120	0.171	0.160	0.221
OB6	0.153	0.184	0.186	0.196	0.166
OB7	0.150	0.176	0.194	0.178	0.141
OB8	0.140	0.170	0.191	0.157	0.158

Covariances

	EN1	EN4	EN5	EN6	CB4

EN1	0.555				
EN4	0.234	0.619			
EN5	0.225	0.349	0.535		
EN6	0.265	0.236	0.268	0.918	
CB4	0.107	0.072	0.089	0.103	0.658
CB5	0.136	0.113	0.094	0.155	0.289
CB6	0.172	0.103	0.110	0.132	0.242
CB7	0.145	0.163	0.128	0.157	0.282
CB8	0.152	0.148	0.164	0.211	0.293
RE1	0.255	0.181	0.234	0.190	0.118
RE2	0.179	0.156	0.182	0.132	0.117
RE3	0.200	0.155	0.193	0.144	0.120
RE4	0.214	0.181	0.216	0.195	0.066
RE7	0.254	0.201	0.227	0.235	0.108
SU1	0.170	0.127	0.171	0.284	0.067
SU5	0.161	0.157	0.147	0.212	0.097
SU6	0.162	0.148	0.163	0.261	0.088
SU7	0.146	0.227	0.217	0.205	0.074
OB2	0.176	0.170	0.187	0.170	0.164
OB4	0.200	0.143	0.180	0.234	0.149
OB6	0.225	0.173	0.162	0.192	0.124
OB7	0.246	0.168	0.192	0.265	0.130
OB8	0.255	0.185	0.162	0.271	0.039

Covariances

	CB5	CB6	CB7	CB8	RE1
CB5	0.734				
CB6	0.376	0.646			
CB7	0.354	0.427	0.757		
CB8	0.295	0.376	0.496	0.811	
RE1	0.156	0.205	0.220	0.245	0.650
RE2	0.169	0.208	0.186	0.191	0.339
RE3	0.156	0.210	0.162	0.200	0.344
RE4	0.102	0.151	0.147	0.165	0.355
RE7	0.127	0.168	0.189	0.223	0.298
SU1	0.153	0.164	0.159	0.183	0.215
SU5	0.126	0.166	0.123	0.136	0.143

SU6	0.159	0.202	0.134	0.175	0.143
SU7	0.112	0.117	0.117	0.131	0.178
OB2	0.188	0.235	0.216	0.266	0.285
OB4	0.210	0.222	0.216	0.273	0.242
OB6	0.213	0.226	0.201	0.238	0.275
OB7	0.210	0.179	0.150	0.204	0.245
OB8	0.195	0.187	0.120	0.173	0.272

Covariances

	RE2	RE3	RE4	RE7	SU1
RE2	0.424				
RE3	0.284	0.520			
RE4	0.276	0.310	0.534		
RE7	0.227	0.254	0.260	0.544	
SU1	0.108	0.174	0.194	0.208	0.960
SU5	0.146	0.129	0.120	0.125	0.317
SU6	0.136	0.163	0.174	0.136	0.437
SU7	0.147	0.139	0.144	0.158	0.266
OB2	0.242	0.236	0.210	0.205	0.174
OB4	0.204	0.219	0.197	0.187	0.208
OB6	0.210	0.235	0.212	0.265	0.204
OB7	0.173	0.211	0.210	0.246	0.264
OB8	0.175	0.214	0.236	0.242	0.267

Covariances

	SU5	SU6	SU7	OB2	OB4
SU5	0.612				
SU6	0.378	0.775			
SU7	0.237	0.327	0.590		
OB2	0.157	0.157	0.165	0.500	
OB4	0.158	0.200	0.145	0.294	0.537
OB6	0.184	0.198	0.156	0.258	0.288
OB7	0.188	0.222	0.149	0.225	0.290
OB8	0.189	0.229	0.147	0.206	0.245

Covariances

	OB6	OB7	OB8
OB6	0.638		
OB7	0.376	0.755	
OB8	0.336	0.459	0.761
